# Intraluminal 10-0 Nylon Stenting in PRESERFLO™ MicroShunt Surgery for Pseudoexfoliation Glaucoma

**DOI:** 10.3390/jcm14176224

**Published:** 2025-09-03

**Authors:** Miranda Gehrke, Leonie F. Keidel, Lara Buhl, Siegfried G. Priglinger, Marc J. Mackert

**Affiliations:** Department of Ophthalmology, LMU University Hospital, LMU Munich, Mathildenstr. 8, 80336 Munich, Germany; miranda.gehrke@med.uni-muenchen.de (M.G.); leonie.keidel@med.uni-muenchen.de (L.F.K.); lara.buhl@med.uni-muenchen.de (L.B.); siegfried.priglinger@med.uni-muenchen.de (S.G.P.)

**Keywords:** glaucoma, pseudoexfoliation, MicroShunt, nylon stenting, hypotony

## Abstract

**Background/Objectives:** Early postoperative hypotony and complications like choroidal detachment can occur after Preserflo MicroShunt (MS) implantation in patients with pseudoexfoliation glaucoma (PEXG). To prevent these risks, outflow from the microshunt tube can be reduced by implementing a nylon stent. This study aims to evaluate the impact of intraluminal stenting of the MS during the first four months after surgery. **Methods:** This retrospective study of 43 eyes investigated the incidence of intraocular hypotony in PEXG patients undergoing MS implantation with (*n* = 23) or without (*n* = 20) intraluminal stenting using a 10.0 nylon suture. The follow-up period was four months after surgery. **Results:** Our results demonstrated that intraluminal stenting significantly reduced the incidence of postoperative complications related to hypotony. Notably, no cases of choroidal detachment occurred in the nylon-stenting group (nsMS) compared to 30% (6 eyes) in the MS-only group (*p* = 0.0064). The hypotony rates between the nsMS (21.74%, 5 eyes) and the MS-only group (40%, 8 eyes) did not significantly differ (*p* = 0.3184). Both groups experienced significant reductions in intraocular pressure (*p* < 0.001) and a decrease in the number of antiglaucomatous medications (*p* < 0.001) up to four months after surgery. **Conclusions:** The use of an intraluminal stent (10.0 nylon suture) during MS implantation may be a promising strategy to reduce the risk of hypotony-related complications, particularly choroidal detachment, in patients with PEXG.

## 1. Introduction

PRESERFLO™ MicroShunt (MS) (Santen, Osaka, Japan) is a filtering procedure that is increasingly used to treat primary open-angle glaucoma (POAG). It is an 8.5 mm long synthetic polymer (SIBS—poly(styrene-block-isobutylene-block-styrene)) tube with a 70 µm diameter lumen, which after implantation allows fluid to flow directly from the anterior chamber of the eye to a filtering bleb underneath the conjunctiva [1]. It has been shown that MicroShunt (MS) effectively achieves low target intraocular pressure (IOP) levels in moderate to severe glaucoma, while reducing or eliminating the need for multiple glaucoma medications [1,2,3,4]. Compared to traditional filtering surgeries like trabeculectomy or glaucoma drainage devices (Ahmed Glaucoma Valve or Baerveldt Glaucoma Implant), MicroShunt (MS) is less invasive, poses a lower risk of complications, and has faster visual rehabilitation [1,2,3].

However, managing patients with pseudoexfoliation glaucoma (PEXG) presents considerable challenges. PEXG, characterized by the deposition of amyloid on the lens, iris, and trabecular meshwork, is often more aggressive and tends to progress rapidly. The extent of anatomical changes due to PEX can be assessed based on slit-lamp findings, like exfoliation load, angle involvement, and zonular instability [5,6,7]., whereas the severity of PEXG is commonly assessed using mean deviation (MD) from visual field testing. These anatomical changes are clinically relevant, as they could potentially influence surgical outcomes. Unfortunately, detailed grading of PEX severity was not available in our retrospective dataset. Due to these changes, PEXG patients are known to be more susceptible to postoperative complications, particularly intraocular hypotony [3,5]. This can subsequently result in severe conditions such as choroidal detachment, hypotony maculopathy, bleeding, and, in some cases, irreversible vision loss [3]. MicroShunt (MS) has been shown to be equally effective in lowering IOP and reducing glaucoma medication in patients with PEXG, with outcomes comparable to those in primary open-angle glaucoma (POAG) [8]. Furthermore, MicroShunt (MS) has been found to be non-inferior to trabeculectomy surgery (TET) in PEXG patients [3].

To minimize the risk of early postoperative hypotony and its complications, non-resorbable sutures can be used as intraluminal stents to ensure controlled drainage of aqueous humor and thus reduce the risk of excessively low IOP and complications such as choroidal detachment [9,10]. Beyond stabilizing IOP, intraluminal stenting also offers several additional benefits. By ensuring a steady, controlled flow of aqueous humor, it promotes proper healing around the shunt and reduces inflammation. Moreover, suture stents are easy to manage during surgery and can be removed at the slit lamp without the need for additional surgery [9,11,12].

This study aims to evaluate the impact of intraluminal stenting with a 10-0 nylon suture in patients with PEXG undergoing MicroShunt (MS) surgery.

The primary goal is to determine whether this technique reduces the incidence of postoperative hypotony and its complications, such as choroidal detachment and vision-threatening hypotony maculopathy. In addition, the study will assess the effect of intraluminal stenting on IOP control, IOP-lowering medication use, time to stent removal, treatment failure, visual acuity, visual field progression, and adverse events during the first four months after surgery.

## 2. Materials and Methods

### 2.1. Study Design

This was a single-center retrospective case-control study including all consecutive patients who underwent MicroShunt (MS) implantation with or without intraluminal stenting at the Department of Ophthalmology, LMU University Hospital, for uncontrolled PEXG between July 2019 and February 2020, and December 2023 and July 2024.

Clinical data was collected according to the principles of the Declaration of Helsinki and approved by the local regulatory ethics committee (Nr. 25-0296). Written informed consent was obtained from all patients.

### 2.2. Study Population

All participants were diagnosed with PEXG that was not adequately controlled with maximum tolerated medical therapy or was progressing despite treatment. Patients who were candidates for glaucoma surgery were eligible for inclusion.

Due to the occurrence of low postoperative IOP following MicroShunt (MS) surgery in patients with PEXG, intraluminal stenting of the MicroShunt (MS) using a 10-0 nylon suture was introduced as a modification. This retrospective analysis included patients who were divided into two groups based on the surgical technique: those who received MicroShunt (MS) implantation with a 10-0 nylon stent (nsMS group) and those who received MicroShunt (MS) without the stent (MS-only group). Due to the retrospective design of the study, there is a potential risk of selection bias, as the decision to use a stent was not randomized but based on individual clinical judgment. Only standalone procedures were performed.

### 2.3. Study Measurements

Postoperative visits followed a standardized regimen. Patients remained in the hospital for two days after surgery, with subsequent follow-up visits at intervals of one week to four weeks, six to eight weeks, and three to four months after surgery.

Before surgery, Visual Field 30-2 testing (Humphrey Field Analyzer 3; Carl Zeiss AG, Oberkochen, Germany) was conducted. Baseline demographic data (age, sex, lens status, spherical refraction, glaucoma medications) were also recorded. For each visit, the patients underwent a comprehensive ophthalmic evaluation, including slit lamp biomicroscopy, fundoscopy, and Goldmann applanation tonometry.

### 2.4. Surgical Success and Failure

In accordance with the World Glaucoma Association’s Guidelines on the Design and Reporting of Glaucoma Surgical Trials [13], failure was defined by any of the following criteria:

1. IOP greater than 17 mmHg or less than 5 mmHg on two consecutive visits.
2. Less than 20% reduction in IOP from baseline on two consecutive visits.
3. The need for surgical revision or reoperation.
4. Loss of light perception.

The first and second postoperative days were excluded from the assessment of surgical success. Needling was not classified as a failure. Complete success was defined as meeting no failure criteria at four months without medication, while qualified success was defined as meeting no failure criteria at four months with medication.

### 2.5. PRESERFLO™ MicroShunt Implantation

**Figure 1:**
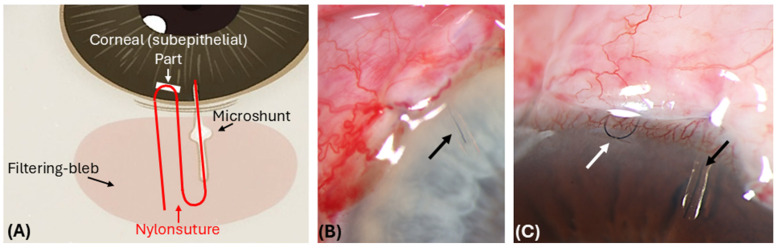
Postoperative (1 day) images showing the position of the 10.0 nylon suture inside the PreserFlo MicroShunt tube. (**A**) Schematic drawing of the placement of the 10.0 nylon suture inside the lumen and intracorneally (U-loop) (in red). (**B**) After implantation of the MicroShunt (MS) tube, a 10-0 nylon suture is advanced to the inner end of the tube (black arrow). (**C**) Then, a 10.0 nylon suture U-loop is embedded subepithelially in the area of the limbal cornea (white arrow), which enables easy postoperative access.

MicroShunt (MS) implantation with or without stenting with a 10.0 suture was performed. Initially, a corneal traction suture was placed with 6-0 silk. A conjunctival limbal incision was made in the superonasal or superotemporal quadrant of the eye, Tenon’s capsule and conjunctiva were dissected, and episcleral tissue was removed. Three sponges soaked in Mitomycin C 0.02% (0.2 mg/mL) were applied to the subtenon space for 2 min, then thoroughly rinsed with balanced salt solution. Afterwards, a 1 mm lance was used to create a scleral tunnel 3 mm from the limbus. A 25-gauge needle was then used to create a path between the iris and cornea, through which the MicroShunt (MS) was inserted in the anterior chamber. The MicroShunt (MS) was irrigated with balanced salt solution (BSS) to verify its function. Nylon stenting was carried out similarly to what has previously been described by Luke et al. [14]: One end of the nylon thread was inserted into the lumen of the MicroShunt (MS). An intrastromal corneal incision was made close to the limbus using a 15-degree knife. The other end of the nylon thread was then embedded in this corneal incision (Figure 1). This step was skipped in the control group. The conjunctiva and Tenon’s capsule were finally placed over the shunt and attached to the limbus with 9-0 Vicryl sutures. A peribulbar injection of 4 mg dexamethasone was applied to reduce postoperative inflammation, followed by topical dexagentamicin ointment, and placement of an eye patch. The procedure was performed under local anesthesia (retrobulbar block) or general anesthesia.

### 2.6. Post-Operative Management

#### 2.6.1. Medication

Following surgery, a comprehensive care plan was implemented to ensure optimal healing and vision recovery. The specific treatment plan for each patient may vary based on individual factors such as IOP and bleb failure.

In general, postoperative management involved the following:

After surgery, the patients discontinued their previous glaucoma medications. To reduce inflammation and promote healing, a topical anti-inflammatory medication regimen (Prednisolone acetate 1% (10 mg/mL) eyedrops) was initiated. While first applied every hour, the dosage was then gradually tapered off every week. To prevent infection, a topical antibiotic (Levofloxacin eyedrops) was administered for a week.

The healing process, the state of the filtering bleb, and IOP were continuously monitored after surgery.

In case of low IOP (<5 mmHG) after surgery, atropine 0.5% eyedrops were started and continued until IOP was above 5 mmHG. IOP-lowering medications may be reintroduced or continued as necessary to maintain target IOP levels. The decision to adjust or discontinue these medications was made by the treating ophthalmologist based on the patient’s individual needs and response to surgery.

#### 2.6.2. Intraluminal Stent Removal

The criterion for stent removal was an IOP of over 15 mmHg. This criterion was based on the surgeons’ clinical experience, aiming to balance the risk of early hypotony and high IOP values. The intraluminal stent removal was performed at the slit lamp under topical anesthesia by grasping the corneal end of the 10-0 nylon suture at the level of the cornea with forceps and pulling it out of the lumen.

#### 2.6.3. Needling and Bleb Revision

Depending on the scarring of the bleb and IOP, 5-Fluorouracil (5-FU) was injected subconjunctivally near the bleb at the examiner’s discretion. If bleb failure or other complications occurred, additional surgical interventions like needling or surgical revision were required. Needling and surgical revision were performed under sterile conditions in the operating room.

Needling was performed in cases of elevated IOP with a scarred and flat bleb. After topical anesthesia, lidocaine was injected under the conjunctiva near the bleb. A 30- or 27-gauge needle was then used to gently break down adhesions in the subconjunctival space both above and below the MicroShunt (MS) with controlled sweeping movements. Finally, 0.1 mL of 5-Fluorouracil (5-FU) was injected into the subconjunctival space, close to the bleb.

If needling failed to lower IOP or scarring in the bleb reappeared, surgical revision was performed. Tenon and conjunctiva were carefully dissected and scar tissue surrounding the implant was removed. Mitomycin C (0.02%; 0.2 mg/mL) was applied using a soaked sponge, which was left in place for two minutes. After removing the sponge, the eye and implant were thoroughly rinsed with BSS. The conjunctiva and Tenon’s capsule were finally placed over the shunt and attached to the limbus with 9-0 Vicryl sutures. The procedure concluded with a peribulbar injection of 4 mg dexamethasone, topical dexagentamicin eyedrops, and an eye patch. Postoperative treatment followed the same protocol as after the initial implantation.

#### 2.6.4. Outcome Measures

The primary outcome measure of the study is IOP and assessment of hypotony-related postoperative complications up to four months after surgery. Secondary outcome measures include the number of IOP-lowering medications and visual acuity, measured as best corrected visual acuity (BCVA) in Snellen. Snellen visual acuity measurements were converted to logMAR equivalents for analysis. Additional measures include the incidence of severe adverse events such as blebitis, endophthalmitis, corneal decompensation, retinal detachment, suprachoroidal hemorrhage, and wipe-out with complete vision loss.

#### 2.6.5. Statistical Analysis

To evaluate potential associations between baseline demographic parameters and intraluminal stent use, multivariable regression analysis was performed with Bonferroni correction. Normal distribution of data was assessed using the Shapiro–Wilk test. Categorical variables like clinical and demographic data were presented as absolute numbers and percentages. Fisher’s exact test was used for categorical variables, as appropriate. To compare means and proportions between the groups, Mann–Whitney U test was used as data was non-normally distributed. For a comparison of more than two groups, repeated measures ANOVA was used. Log-rank test was used to compare survival distributions. A post hoc power analysis was conducted to determine the minimum detectable effect size at a power of 0.8. A *p*-value of less than 0.05 was considered statistically significant. Confidence intervals (CIs) were calculated using a two-tailed method at a 95% confidence level. Statistical analyses and graph plotting were performed using Graphpad Prism for Windows (version 10.4.1; USA).

## 3. Results

### 3.1. Baseline Characteristics

This study included a total of 43 eyes, with 23 eyes in the MicroShunt with nylon stenting (nsMS) group and 20 eyes in the MicroShunt (MS-only) group. Baseline demographic data of both groups are presented in Table 1.

The patients ranged from 60 to 93 years old, with a median age of 75.7 ± 8.2 years. Both groups had similar age (*p* = 0.98, Mann–Whitney U test) and gender (*p* = 0.07, Fisher’s exact test). Preoperative intraocular pressure (*p* = 0.3289, ANOVA) and BCVA (*p* = 0.3129, Mann–Whitney U test) were comparable between the two groups. The nsMS group showed greater visual field loss (mean deviation (MD) of the nsMS group was −14.66 decibel (dB) ± 9.28 dB, whereas mean MD of MS-only group was −7.65 dB ± 5.76 dB (*p* = 0.01, Mann–Whitney U test)).

The same number and types of intraocular pressure-lowering medications were used by both groups preoperatively (*p* = 0.79, Mann–Whitney U test). Meanwhile, lens status (*p* = 0.35, Fisher’s exact test), corneal thickness (*p* = 0.18, Mann–Whitney U test), spherical refraction (*p* = 0.91, Mann–Whitney U test), and the history of prior glaucoma surgeries (*p* ≥ 0.9999 Fisher’s exact test) were comparable.

Multivariable regression analysis found that among demographic factors, visual field loss, expressed as MD, was significantly associated with stent use (*p* = 0.007, Bonferroni-corrected multivariable regression analysis). Other factors, including age, sex, pseudophakia, baseline visual acuity, IOP, and number of medications, did not show statistically significant associations.

### 3.2. Primary Outcome Measures

**Figure 2:**
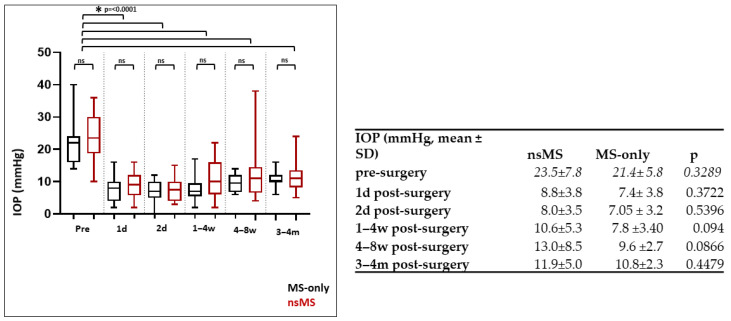
Development of IOP (mmHg) in the MS-only and nsMS groups from preoperative levels to the four-month follow-up visit (mean ± SD). IOP decreased significantly in both the MS-only and the nsMS group from before surgery to 4 months postoperatively (*p* < 0.001, Mann–Whitney U test). The reduction in IOP did not differ between the groups at all measured times up to 4 months postoperatively (d = day; w = week; m = month; ns = not significant).

At all measured timepoints up to 4 months (Figure 2), intraocular pressure (IOP) decreased significantly in the MS-only group from a preoperative mean of 21.4 mmHg (±5.76) [CI 95%: 18.70 to 24.10 mmHg] to postoperative means at each timepoint: 1 day (7.4 mmHg ± 3.8) [CI 95%: 5.64 to 9.16 mmHg], 2 days (7.05 mmHg ± 3.2) [CI 95%: 5.57 to 8.53 mmHg], 1–4 weeks (7.8 mmHg ± 3.4) [CI 95%: 6.18 to 9.35 mmHg], 6–8 weeks (9.6 mmHg ± 2.7) [CI 95%: 8.33 to 10.87 mmHg], and 3–4 months (10.81 mmHg ± 2.28) [CI 95%: 9.74 to 11.88 mmHg] (*p* < 0.001, Mann–Whitney U test).

Also, in the nsMS group, IOP declined from a preoperative mean of 23.54 mmHg (±7.81 mmHg) [CI 95%: 20.16 to 26.92 mmHg] to postoperative means at 1 day (8.8 mmHg ± 3.8 mmHg) [CI 95%: 7.18 to 10.49 mmHg], 2 days (8.0 mmHg ± 3.5) [CI 95%: 6.50 to 9.50 mmHg], 1–4 weeks (10.6 mmHg ± 5.3) [CI 95%: 8.32 to 12.88 mmHg], 6–8 weeks (13.0 mmHg ± 8.5) [CI 95%: 9.28 to 16.62 mmHg], and 3–4 months (11.90 mmHg ± 5.01) [CI 95%: 9.74 to 14.07 mmHg]. This reduction was also statistically significant (*p* < 0.001, Mann–Whitney U test) (Figure 2).

After 4 months postoperatively, there was a reduction in the IOP of 49.45% in the nsMS group versus 49.49% in the MS-only group. The IOP reduction to preoperative values did not differ between the groups at all measured timepoints up to 4 months postoperatively. Significance levels are depicted in Figure 2. The achieved powers were calculated to be 0.23 (minimum effect size: 112) at day 1, 0.151 (minimum effect size: 192) at day 2, 0.546 (minimum effect size: 40) at week 1–4, 0.407 (minimum effect size: 58) at week 4–6, and 0.483 (minimum effect size: 47) at month 3–4.

The lowest recorded IOP was 0 mmHG in the MS-only group and 2 mmHg in the nsMS group. The rate of hypotony was higher in the MS-only group (40%) compared to the nsMS group (21.74%), though this difference did not show to be statistically significant (*p* = 0.3184, Mann–Whitney U test). Achieved power was 0.162 (minimum effect size: 110) (Figure 2).

### 3.3. Medical Therapy

**Figure 3:**
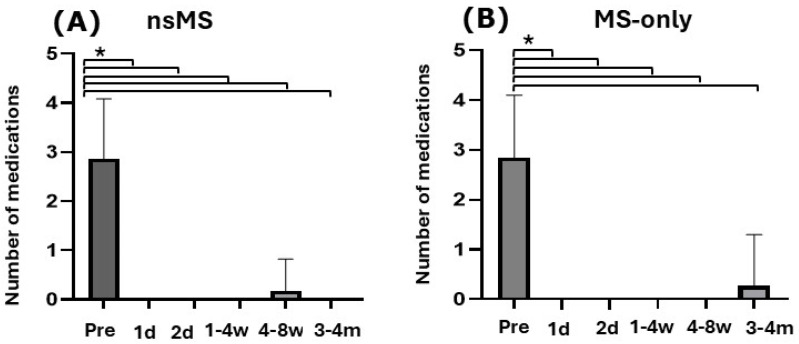
Histogram showing the number of IOP-lowering medications used preoperatively from day 1 to the 4-month follow-up visit (mean ± SD). In both groups, the average number of IOP-lowering medications significantly decreased after 3 to 4 months. (**A**) In the nsMS group antiglaucomatous medication decreased from 2.88 ± 1.19 to 0 (*p* < 0.0001; Fisher’s exact test) (**B**) In the MS-only group antiglaucomatous medication decreased from 2.75 ± 1.2 to 0.25 (*p* < 0.0001; Fisher’s exact test) (Pre = preoperatively; d = day; w = week; m = month; * *p* ≤ 0.0001)).

In both groups, the average number of IOP-lowering medications significantly decreased in the nsMS group from 2.88 ± 1.19 to 0 (*p* < 0.0001; Fisher’s exact test) and in the MS-only group from 2.75 ± 1.2 to 0.25 (*p* < 0.0001; Fisher’s exact test) after 3 to 4 months. As illustrated in Figure 3, after 3 months, a single eye (4.3%) in the nsMS group needed three IOP-lowering drugs within 4 to 8 weeks. This eye received needling after a week and bleb revision after 2 weeks post-surgery due to bleb failure. IOP-lowering medication was no longer indicated after these procedures.

In the MS-only group, one eye (5%) needed 4 IOP-lowering eyedrops after 4 months, because of progressive visual field defects despite an IOP of 13 mmHG.

There was no significant difference in reduction of medication between the two groups (*p* ≥ 0.9999; Fisher’s exact test). The calculated power for this comparison was 0.062 (minimum effect size: 156).

### 3.4. Intraluminal Stenting

**Figure 4:**
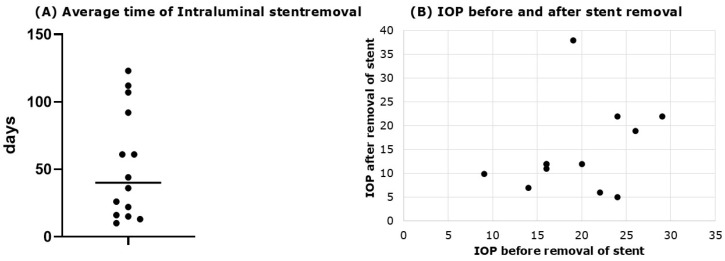
Time of stent removal and effect on IOP. (**A**) Average removal time of intraluminal stent (average removal time of 52.72 ± 40.45 days); (**B**) IOP before and after stent removal. Pre-removal IOP was 18.86 ± 5.72 mmHg, dropping to 14.67 ± 9.31 mmHg (*p* = 0.03836, Mann–Whitney U test).

In 14 eyes (60.87%), the intraluminal stent was removed, with an average removal time of 52.72 ± 40.45 days [CI 95%: 29.35 to 76.09 days] (Figure 4) and a mean IOP reduction of 4.19 ± 3.6 mmHg [CI 95%: 2.11 to 6.27 mmHg] after removal. Pre-removal IOP was 18.86 ± 5.72 mmHg [CI 95%: 15.56 to 22.16 mmHg], dropping to 14.67 ± 9.31 mmHg [CI 95%: 9.29 to 20.05 mmHg] (*p* = 0.038, Mann–Whitney U test) (Figure 4).

### 3.5. Postoperative Complications

Table 2 provides an overview of the postoperative complications in each group. The most prevalent complication was hypotony. Hypotony occurred in 21.74% eyes in the nsMS group (5 eyes) and 40% in the MS-only group (8 eyes), with no statistically significant difference (*p* = 0.3184; Fisher’s exact test). The power reached was calculated to be 0.162 (minimum effect size: 110). All cases of hypotony in the MS-only group occurred early after surgery and resolved within the first 6 weeks. In six eyes in the MS-only group and in one case in the nsMS group, hypotony resolved spontaneously. Others required conservative treatment with topical atropine and/or therapeutic contact lenses (four eyes (80%) in the nsMS group and two eyes (10%) in the MS-only group). In total, 2 cases (10%) in the MS-only group and 0 cases (0%) in the nsMS group required stabilization of the anterior chamber with viscoelastic.

However, the MS-only group experienced significantly more complications related to excessively low IOP. Specifically, 6 eyes (30%) in the MS group without nylon stenting developed choroidal detachment. In contrast, none (0%) of the patients in the nsMS group experienced these complications (*p* = 0.0064, Fisher’s exact test). Achieved power was 0.752 (minimum effect size: 24). Interestingly, five days after removal of the intraluminal stent, one patient experienced intraocular hypotony with choroidal detachment; however, the intraocular hypotony and choroidal detachment resolved spontaneously after the use of topical atropine 1%.

Both groups experienced complications such as Seidel-positive leakage (one in the MS-only group and two in the nsMS group (*p* ≥ 0.9999, Fisher’s exact test)) and a flat anterior chamber (three in the MS-only group and one in the nsMS group (*p* = 0.3235, Fisher’s exact test)). Hyphema was seen in the MS-only group in 4 cases, but nsMS did not experience hyphema (*p* = 0.0393, Fisher’s exact test)

Corneal erosion could be observed in two patients in the MS-only group, whereas it was absent in the nsMS group (*p* = 0.2104, Fisher’s exact test). Both groups experienced one case of corneal edema (*p* ≥ 0.9999, Fisher’s exact test). In none of the groups, macular folds, corneal dellen, implant extrusion, blebitis, or loss of light perception was recorded.

Importantly, no serious adverse events were reported in either group.

The resulting power was calculated to be 0.51 (minimum effect size: 676) for Seidel-positive leakage, 0.111 (minimum effect size: 121) for flat anterior chamber, 0.458 for hyphema (minimum effect size: 37), 0.135 (minimum effect size: 76) for corneal erosion, and 0.093 (minimum effect size: 692) for corneal edema.

### 3.6. Subconjunctival Injection of 5-FU, Needling, and Surgical Revision

In the MS-only group, subconjunctival 5-FU injections were administered 24 times (1.2 injections per case) compared to 40 times (1.74 injections per case) in the nsMS group (*p* = 0.1004, Fisher’s exact test). The number of 5-FU injections did not differ significantly (*p* = 0.117, Fisher’s exact test) between the two groups. The statistical power obtained was 0.170 (minimum effect size: 50).

A single 5-FU injection was sufficient in 11 cases (55%) in the MS-only group and 8 cases (34.78%) in the nsMS group. Two injections were required in 6 cases (30%) in the MS-only group and 12 cases (52.2%) in the nsMS group. Three injections were necessary in 3 cases (13.04%) in the nsMS group. Notably, 3 eyes (15%) in the MS group did not require any postoperative 5-FU injections, whereas every eye in the nsMS group received at least one.

On the second day and the 4- to 8-week mark, the total number of eyes requiring 5-FU injections differed significantly between the groups (*p* = 0.0387 and *p* = 0.050, Fisher’s exact test), with the nsMS group receiving more injections. No significant differences could be observed at other timepoints (*p* ≥ 0.9999, Fisher’s exact test).

Needling was performed when a flat bleb, elevated IOP, or a Tenon’s cyst were present. The total number of eyes requiring needling did not differ significantly between the two groups (*p* > 0.9999, Fisher’s exact test). The calculated power was 0.067 (minimum effect size: 801). At all times, there was no significant difference in needling rates (*p* > 0.9999, Fisher’s exact test).

Early bleb encapsulation requiring needling occurred in 2 cases (10%) in the MS-only group and 2 cases (8.7%) in the nsMS group. In the MS-only group, needling was performed at either day 1 or between 4 and 8 weeks postoperatively. In the nsMS group, needling was performed between 4 and 8 weeks and 3 and 4 months postoperatively.

Within the first week after surgery, two patients in the MS-only group required three interventions (three anterior chamber reformations with Healon (hyaluronic acid sodium 8.5 mg/0.85 mL)), while one patient in the nsMS group required a single intervention (re-suturing of the conjunctiva) (*p* = 0.3475, Fisher’s exact test).

During the four-month follow-up, surgical revision was not needed in the MS-only group (0%), whereas it was necessary in three cases in the nsMS group (13%) (*p* = 0.2464, Fisher’s exact test). Besides, one patient in the MS-only group required rescue surgery with repositioning and suture-fixation of the implant, along with occlusion of the tube using an 8.0 Ethilon suture. Whereas no such procedure was needed in the nsMS group (*p* = 0.4773, Fisher’s exact test).

Further glaucoma surgery was required for one patient in the MS-only group. Four months after initial, complicated surgery and the above-mentioned rescue surgery on the fourth postoperative day as well as unsuccessful needling, cyclophotocoagulation was performed due to scarring of the bleb and conjunctiva as well as underlying systemic comorbitities that limited the feasibility of further glaucoma-valve surgery. Up to the four-month follow-up, no patients in the nsMS group required further surgery (*p* = 0.4773, Fisher’s exact test).

### 3.7. Success Rates

**Figure 5:**
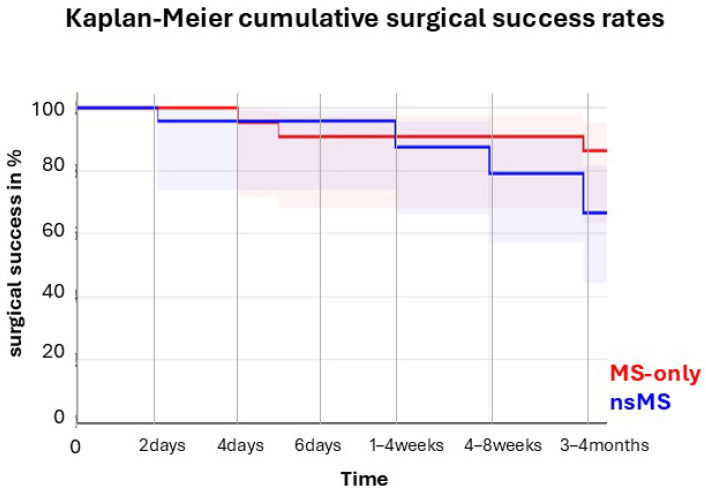
Kaplan–Meier survival curve of cumulative surgical success through four months of follow-up.

In the MS-only group, 85% (17/20) achieved complete success compared to 65.3% (15/23) in the nsMS group (*p* = 0.1396; Log-rank test). Qualified success was achieved in 90% (18/20) of the MS-only group and 82.6% (19/23) of the nsMS group (*p* = 0.1396; log-rank test). Kaplan–Meier survival curves for complete success are shown in Figure 5.

In the nsMS group, three patients (13.0%) experienced an IOP outside the 5–17 mmHg range on two consecutive visits—two after 3–4 months and one after 6 weeks. Moreover, another patient (4.3%) experienced an IOP reduction of less than 20% from baseline to 3–4 months compared to none in the MS-only group.

After four or five days, two patients (10%) in the MS-only group developed postoperative hypotony, requiring AC reformation with Healon. Another patient needed further glaucoma surgery (cyclophotokoagulation) after 4 months. In the nsMS group, one patient required surgery after 2 days (re-suturing of the conjunctiva) due to a Seidel I-positive filtering bleb and three patients required open revision surgery for a scarred bleb after two, four, and seven weeks (13.0%).

## 4. Discussion

Postoperative hypotony is a recognized risk for MicroShunt (MS) surgery. Unlike the Ahmed valve, which regulates aqueous outflow through a built-in resistance mechanism, MicroShunt (MS) is based on the principles of the Hagen–Poiseuille law and the gradual development of outflow resistance caused by fibrosis in the conjunctiva and Tenon’s capsule [15,16]. This risk, along with the potential for sight-threatening complications such as choroidal detachment, macular folds, and suprachoroidal hemorrhage, is further heightened in PEXG patients undergoing MicroShunt (MS) surgery [3,16,17], as in PEXG patients, conjunctival and scleral healing can be impaired by oxidative stress and chronic inflammation due to exfoliative material [17,18]. Additionally, a higher incidence of complications such as zonular dialysis and vitreous loss may further disrupt drainage through the MicroShunt (MS) tube [5,19]. Consequently, to prevent hypotony in PEXG patients, measures like reducing outflow from the MicroShunt (MS) tube using a nylon stent should be considered during surgery.

This retrospective study of 43 eyes investigated the incidence of intraocular hypotony in patients with PEX glaucoma undergoing MicroShunt (MS) implantation with (*n* = 23) or without (*n* = 20) intraluminal stenting with a 10.0 nylon suture. The follow-up period was 4 months after surgery. Although the use of a 10.0 nylon stent for outflow restriction as a treatment for prolonged postoperative hypotony has been described previously [10,11,12], this, to our knowledge, is the first study to evaluate the efficacy of MicroShunt (MS) flow restriction in PEX glaucoma eyes and its preventive impact on early hypotony rates and associated complications. The two groups were demographically comparable, except for significantly more advanced visual field defects (expressed as mean deviation (MD)) in the nsMS group (*p* = 0.01). Moreover, MD significantly predicted the likelihood of stent implantation (*p* = 0.007). This may be attributed to consistent clinical outcomes with MicroShunt (MS) implantation in PEXG cases, which has encouraged its use in more advanced stages of PEXG. However, it also suggests a potential treatment bias, whereby surgeons may have been more inclined to select patients with more severe disease for intraluminal stenting.

Previously, it was demonstrated that MicroShunt (MS) implantation is effective for PEXG, showing non-inferiority compared to trabeculectomy (TET) [3]. However, PEXG patients experienced a higher incidence of hypotony, particularly with anterior chamber shallowing, compared to MicroShunt (MS) surgery performed in patients with primary open-angle glaucoma (POAG) [8,20]. As a preventive measure, intraluminal stents may be used—similar to Baerveldt or Paul glaucoma drainage devices, where such stents are routinely placed and typically removed after 4 to 6 weeks.

Nylon stenting with a 10.0 nylon suture (20 µm diameter) reduces the lumen of the MS (70 µm diameter) by 29% and consequently decreases outflow. That is why lower rates of hypotony were hypothesized in the nsMS group, as demonstrated previously in other cohorts [10,11,12,14].

However, in the present study, no significant difference in early postoperative hypotony rates between the MS-only and nsMS groups could be found. Although the hypotony rate was higher in the MS-only group (40%, 8 eyes) compared to the nsMS group (21.74%, 5 eyes), the difference was not statistically significant (*p* = 0.3184). This lack of difference may be attributed to factors such as the extent of MicroShunt (MS) occlusion or the small sample size, causing this study to be underpowered to rule out a clinically meaningful difference. Moreover, in contrast to Luke et al., Lupardi et al., Miura et al., and Verma-Fuehring et al. [10,11,12,14], our patient population consisted solely of PEXG patients, who might respond differently to intraluminal stenting. PEXG is known to exhibit fluctuating IOP values and is generally more aggressive, with a higher risk of postoperative hypotony [17]. As mentioned before, the size of the suture could play a role in IOP control. Here, we used a 10.0 nylon suture, which is about 20 µm in diameter and occludes approximately 29% of the MicroShunt (MS) (lumen: 70 µm diameter). In contrast, Luke et al. [14] used a thicker 8-0 polyamide suture, which has a larger diameter (40 µm) and occludes up to 50% of the MicroShunt (MS) lumen. The thicker suture may be more effective in reducing postoperative hypotony. However, it is important to distinguish uncomplicated hypotony—which typically resolves spontaneously within 4–6 weeks—from clinical hypotony associated with complications such as choroidal detachment and anterior chamber shallowing.

Although the overall difference in the rate of hypotony did not reach statistical significance, clinical hypotony was significantly lower in the nsMS group. Notably, no cases of choroidal detachment, a serious sight-threatening complication, occurred in the stenting group compared to 30% (6 eyes) in the MS-only group (*p* = 0.0064). Interestingly, hyphema occurred less frequently in the nsMS group (*p* = 0.0393), possibly due to a lower incidence of clinical hypotony, as reflux bleeding is associated with excessively low postoperative IOP levels. Other complications, such as corneal edema (*p* > 0.9999), corneal erosion (*p* = 0.2104), and shallow anterior chamber (*p* = 0.3235), remained comparable between the groups. This could suggest that nylon stenting does not cause additional trauma.

While the intraluminal stent provided fewer hypotony-related complications, it did not significantly affect long-term IOP control or the need for glaucoma medications. Both groups had comparable reductions in anti-glaucoma eyedrop usage (*p* = 0.999), and surgical success (*p* = 0.1396) was similar. Needling rates in our study were the same in both groups (Needling: MS-only group 10% and nsMS-group 8.7%) and in line with previously published studies. Thus, needling rates of 5% to 19% were reported after one year of follow-up [14,21]. Additionally, the number of eyes (*p* = 0.117) needing 5-FU injections and total number of 5-FU injections did not differ significantly (*p* = 0.10). These findings suggest that the 10.0 intraluminal stent appears to effectively stabilize IOP without compromising MicroShunt (MS) filtration efficacy at up to 4 months.

However, four patients in the nsMS group experienced IOP levels greater than 17 mmHg or a reduction of less than 20%. This could be attributed to the intraluminal stent not yet being removed; therefore, optimal timing for stent removal is essential to achieve better long-term IOP control. Nevertheless, to avoid hypotony after early suture removal, we recommend pulling out the nylon suture no earlier than 4 weeks, once the drainage bleb has formed. If necessary, in the case of rising IOP, topical anti-glaucoma medications, preferably beta-blockers or carbonic anhydrase inhibitors, should be used during this period. In cases where IOP remains consistently below 15 mmHg, the intraluminal stent is retained long-term. This approach is based on findings from previous studies [10,11,12,14], which have not demonstrated any disadvantages associated with stent retention. Maintaining the stent could help reduce the risk of delayed-onset hypotony, which can occur even after initially stable postoperative outcomes. However, a longer follow-up period would be required to thoroughly assess the long-term safety and efficacy of this approach.

However, patients with nylon stenting may be at an increased risk of complications, such as endophthalmitis. This risk is thought to arise from the stent serving as a conduit between the anterior chamber and the subconjunctival space [12,22]. None of our patients experienced this complication, and moreover, previous studies have reported no cases of infection following intraluminal stent placement [10,11,12,14]. To minimize the risk of infection, we adopted a technique first described by Luke et al. [14], in which the nylon suture is embedded into the cornea (Figure 1). Burying the external end of the suture in a corneal groove simplifies postoperative management, lowers the risk of bacterial invasion, reduces the need for invasive removal procedures, minimizes accidental suture pullout, and improves patient comfort.

In conclusion, this study provides preliminary evidence that intraluminal stenting with a 10.0 nylon suture during MicroShunt (MS) implantation may be a promising strategy to reduce the risk of hypotony-related complications in PEXG patients. The technique is minimally invasive and technically straightforward and integrates seamlessly into existing surgical workflows. However, this study has several limitations. Its retrospective design may introduce selection bias and limit control over data collection, as the decision to use a stent may have been influenced by clinical judgment, potentially favoring stent placement in higher-risk cases. Indeed, glaucoma severity, as measured by mean MD, was significantly associated with stent implantation (*p* = 0.007), suggesting a possible treatment bias in which surgeons preferentially selected patients with more advanced PEXG for intraluminal stenting. This bias may confound interpretation of treatment outcomes, as more severe baseline disease could lead to different postoperative trajectories. Glaucoma severity was assessed using visual field MD as recommended by the World Glaucoma Association’s Guidelines on the Design and Reporting of Glaucoma Surgical Trials. However, we did not apply a PEX-specific grading system that considers factors such as exfoliation load, angle involvement, or zonular instability. This limits our ability to address disease heterogeneity within the PEXG population. Prospective studies with standardized criteria for stent selection are needed to reduce bias and more accurately assess the efficacy of stenting across varying disease severities.

Lastly, the relatively small sample size (*n* = 43) limits the statistical power and generalizability of our findings. Several comparisons did not reach statistical significance and should therefore be interpreted with caution. Post hoc power analysis indicated insufficient power to detect small to moderate effect sizes, meaning that the study may not have been adequately powered to rule out clinically relevant differences. Larger, well-powered studies are warranted to validate and expand upon these results.

## Figures and Tables

**Table 1:** Summary of demographic characteristics of study patients at baseline in the MS-only and nsMS groups.

	nsMS (10.0) (*n* = 23)	MS-Only (*n* = 20)	*p*
Age (y, mean ± SD)	75.54 ± 7.79	75.95 ± 8.85	0.98 ^a^
Female [*n* (%)]	8 (34.8%)	13 (65%)	0.0690 ^b^
Pseudophakic [*n* (%)]	14 (60.9%)	15 (75%)	0.3528 ^b^
Bilateral cases [(%)]	2 (9%)	2 (9%)	>0.9999 ^b^
Combined surgery [(%)]	0 (0%)	1 (5%)	0.4651 ^b^
Baseline IOP (mmHG)	23.54 ± 7.81	21.4 ± 5.76	0.3289 ^c^
Baseline MD (mean ± SD)	−14.54 ± 9.38	−7.65 ± 5.76	0.01 ^a^
Baseline BCVA (logmar, mean ± SD)	0.30 ± 0.22	0.18 ± 0.22	0.3129 ^c^
Baseline medications (*n*, mean ± SD)	2.88 ± 1.19	2.75 ± 1.29	0.7919 ^a^
CCT (µm, mean ± SD)	532.18 ± 37.17	544.7 ± 52.76	0.18 ^a^

^a^ Mann–Whitney U test; ^b^ Fisher’s exact test; ^c^ repeated measures ANOVA; CCT = central cornea thickness; BCVA = best corrected visual acuity; MD = mean deviation, SD = standard deviation.

**Table 2:** Postoperative complications after MS-only and nsMS surgery.

	nsMS (*n* = 23)	MS-Only (*n* = 20)	*p*
Hypotony (in%)	5 (21.74%)	8 (40%)	0.3184
Choroidal detachment (in%)	(0) 0%	(6) 30%	0.0064
Flat anterior chamber (*n* (%))	(1) 4%	(3) 15%	0.3235
Macular folds	(0) 0%	(0) 0%	
Hyphema	(0) 0%	(4) 20%	0.0393
Corneal complications (*n* (%))	(1) 4%	(3) 15%	0.3235
Corneal dellen (*n* (%))	(0) 0%	(0) 0%	>0.9999
Corneal erosion (*n* (%))	(0) 0%	(2) 10%	0.2104
Corneal edema (*n* (%))	(1) 4%	(1) 5%	>0.9999
Seidel positive (*n* (%))	(2; Seidel I + Seidel II) 9%	(1; Seidel I) 5%	>0.9999
Implant extrusion (*n* (%))	0%	(0) 0%	>0.9999
Blebitis (*n* (%))	0%	(0) 0%	>0.9999
Loss of light perception (*n* (%))	0%	(0) 0%	>0.9999

Data are presented as numbers and percentages of the total number for the respective treatment group (MS-only or nsMS). The nsMS group had significantly lower number of choroidal detachment (*p* = 0.0064, Fisher’s exact test) and hyphema (*p* = 0.0393, Fisher’s exact test). Other complications were similar.

## Data Availability

All relevant data was provided in the manuscript.
